# Isoflurane vs. Propofol Sedation in Patients with Severe Stroke: A Clinical Proof-of-Concept-Study

**DOI:** 10.3390/jcm14051594

**Published:** 2025-02-26

**Authors:** André Worm, Christian Claudi, Svea R. Braun, Marisa Schenker, Anneke Meyer, Leona Moeller, Ole J. Simon, Lars Timmermann, Anne Mrochen, Norma J. Diel, Martin Juenemann, Hagen B. Huttner, Patrick Schramm

**Affiliations:** 1 Department of Neurology, University Hospital of the Justus-Liebig University, 35392 Giessen, Germany; andre.worm@neuro.med.uni-giessen.de (A.W.); christian.claudi@neuro.med.uni-giessen.de (C.C.); marisa.schenker@med.uni-giessen.de (M.S.); anneke.meyer@med.uni-giessen.de (A.M.); anne.mrochen@neuro.med.uni-giessen.de (A.M.); norma.diel@neuro.med.uni-giessen.de (N.J.D.); martin.juenemann@neuro.med.uni-giessen.de (M.J.); hagen.huttner@neuro.med.uni-giessen.de (H.B.H.); 2 Department of Neurology, University Hospital of the Philipps-University Marburg, 35043 Marburg, Germany; steinmey@med.uni-marburg.de (L.M.); ole.simon@uk-gm.de (O.J.S.); lars.timmermann@uk-gm.de (L.T.)

**Keywords:** neuro critical care, volatile sedatives, stroke, isoflurane, propofol, delirium

## Abstract

**Background**: Severe strokes often require deep sedation, yet the optimal sedation regimen remains unclear. This comparative study compared the efficacy of achieving target sedation depth using inhaled (isoflurane) versus intravenous (propofol) sedation. **Methods**: This prospective, observational, proof-of-concept study was conducted between July 2022 and June 2023 at two University Hospitals with dedicated neurological intensive care units. We included conservatively treated patients with severe space-occupying strokes (ischemic or haemorrhagic) requiring deep sedation. Patients received either inhaled or intravenous sedation. Sedation targets were defined in the morning rounds using the Richmond-Agitation-Sedation-Scale and were assessed at two subsequent time points (7 p.m. and 7 a.m.) during hospital stay. The primary outcome was the number of days where the predefined sedation target was achieved at both time points, comparing between the two sedation regimens. Secondary and safety outcomes included the incidence of delirium, pneumonia, functional outcomes, mortality, and vasopressor doses. **Results**: Seventy-nine patients (age 71 [63–81] years, 31 female) were included. Patients sedated with isoflurane achieved the sedation target significantly more often, with 182/444 (41%) compared to 80/497 (16%) assessments in patients sedated with propofol (RR 1.4; 95%-CI: 1.3–1.6). This effect was consistent across all sedation stages, specifically in the deep sedation targets (RR 1.5; 95%-CI: 1.2–1.9) and no-sedation target (RR 5.1; 95%-CI: 2.8–9.4). Secondary and safety outcomes revealed no significant differences. **Conclusions**: Isoflurane sedation offers a benefit for invasively ventilated stroke patients with respect to sedation targets. Specifically, isoflurane facilitates faster awakening when transitioning from deep sedation to awakening. These data encourage further confirmatory studies for specific stroke-patient groups.

## 1. Introduction

There is a large body of level I evidence regarding specific endovascular and surgical treatments for stroke patients, especially thrombectomy, decompressive surgery or minimally invasive strategies to reduce mass effects [1,2]. Contrarily, the so called “basic management” of stroke patients requiring critical care, including sedation strategies, is essentially scarce [3]. Yet, critical care patients with neurovascular disease, notably ischemic and haemorrhagic stroke, often require prolonged sedation and ventilation [4]. The latter is mainly necessary in cases of a space-occupying mass effect of the lesions to reduce neuronal activity, mitigating vegetative stress and blood pressure fluctuations [5]. Additionally, secondary complications like severe ventilator-associated pneumonia may further impede reducing sedation and rousing those patients [6]. In parallel, guidelines recommend regular neurological examinations, which, in daily routine, represent a challenge given the suggested sedatives, i.e., propofol and midazolam [7]. Both drugs show relevant context-sensitive half-times (CSHT) which result in prolonged awakening and additional distinct side effects, including delirium and toxicity [8].

Volatile anaesthetic agents, in particular isoflurane, have a relatively short CSHT due to minimal metabolism required to process the drug compared to propofol or midazolam [8,9]. In light of numerous further advantages of volatile anaesthetics over intravenous sedatives [10], these drugs are being widely used for short-term sedation of non-neurological patients [11]. However, patients usually did not require an initial period of deep sedation and the results of the studies are therefore not applicable to stroke patients. In stroke patients, there have been only a few observational studies using specific devices for inhaled sedation, and these studies mainly addressed associations on cerebral oxygenation, regional cerebral blood flow and intracranial pressure [12]. However, whether volatile anaesthetics could offer advantages over propofol for sedation in stroke patients remains largely unexplored.

The use of volatile anaesthetics for managing prolonged sedation in stroke patients may represent an elegant approach achieving both deep sedation and timely neurological examination, thus allowing a more titrated sedation management for achieving certain target Richmond-Agitation-Sedation-Scale (RASS) scores [13]. To address this unstudied issue, we here conducted a pragmatic comparative clinical proof-of-concept-study exploring the associations of isoflurane versus propofol with outcomes in critically ill stroke patients.

## 2. Materials and Methods

### 2.1. Study Design and Population

This study was a prospective observational, bicentric, comparative, clinical, proof-of-concept, pilot cohort study in adults with severe stroke. Between July 2022 and June 2023, we screened all consecutive adult patients admitted with ischemic or haemorrhagic stroke to the dedicated neurocritical care units of the Departments of Neurology at the University Hospitals of Giessen and Marburg, Germany. We included patients who met the criteria for prolonged sedation and mechanical ventilation (>72 h) due to severe stroke with proven regional or global space-occupying effects.

The diagnosis of cerebral ischemia (AIS) or intracerebral haemorrhage (ICH) was made in accordance with established criteria [14,15]. Secondary transfers from rural hospitals > 24 h of symptom onset were not included in the study, with the focus being on acute stroke patients with AIS who were undergoing reperfusion therapies, namely intravenous thrombolysis using tissue-type plasminogen activator (rt-PA), mechanical thrombectomy or both [16]. Moreover, the decision to administer sedation and mechanical ventilation was based on previously defined criteria [14,15]. An infarct size exceeding 50% of the middle cerebral artery (MCA) or cerebellum territory was assumed to indicate regional or global space-occupying effects [17]. In patients with ICH, a hematoma volume > 30 mL, assessed by the ABC/2 method [18], was considered as a space-occupying mass. Women who were pregnant or lactating, as well as patients with known intolerance or contraindication to the anaesthetics used, were excluded a priori. In the final analysis, all patients who did not provide consent (legal representative) within 24 h or who withdrew consent during their hospital stay were excluded.

### 2.2. Group Allocation

A total of 18 (2 × 9) neuro-intensive beds were available at both sites during the one-year study period. All beds were equipped for mechanical ventilation, whereas nine beds were additionally equipped with a gas delivery system. Thus, allocation to a regular intensive care bed versus one with gas delivery system was performed on a 1:1 ratio based on respective availability upon patient admission. In situations with availability of both types of beds, we a priori determined to allocate the patient to a gas-capable bed and subsequently allocated consecutive patients to regular beds until the 1:1 ratio was restored. This allocation mechanism was carried out by the shift leader nurse of the respective shift at the time of patient’s admission, who at the timepoint of randomization was blinded to type and severity of patients’ disease, age, comorbidities, or further parameters.

### 2.3. Patients’ Characteristics

The baseline patients’ characteristics including age, sex, body mass index (BMI), the modified Rankin scale before stroke (pre-mRS), sequential organ failure assessment (SOFA) score, the national institute of health stroke scale (NIHSS) at admission and at discharge from the intensive care unit (ICU) and various laboratory parameters on admission and during hospital stay were documented and analysed.

### 2.4. Management of Sedation

Administration of sedation was conducted either with isoflurane (inhaled sedation (isoflurane-) group) or propofol (intravenous sedation (IV-) group), utilized according to their respective application descriptions and institutional protocols [19]. Isoflurane administration was performed using the Mirus™ vaporizer (TIM Medical, Koblenz, Germany) [20] and controlled by the minimal alveolar concentration (MAC). IV administration was performed using continuous application of weight-adjusted propofol. Patients in both groups were sedated according to a defined standard operating procedure (Supplementary Figure S1) and received analgesia with continuous application of appropriate dosages of sufentanil. The administered amount of isoflurane was recorded as the daily average MAC. For propofol and sufentanil, the administered dose per hour and per kilogram of body weight was averaged over a day.

During the first 72 h of intensive care, a deep sedation strategy, i.e., target RASS score between −5 to −4, was carried out, reducing cerebral metabolism and vegetative stress, followed by a moderate sedation strategy, i.e., RASS score −3 to −1, as described previously, for a period of 3–7 days [21]. The decision as to whether sedation was reduced during this period was based on repeated follow up CT scans every 48 ± 24 h to monitor mass effect with continued sedation if considered required [21]. After this period, the sedation was stopped, and a RASS of 0 was targeted. Data were recorded up to either extubation or transfer to another hospital or up to 30 days in those patients who remained that entire period in one of the neurocritical care units.

### 2.5. Primary Outcome

The primary outcome measure was achieving the sedation target, assessed by RASS, and scored as days with versus without fulfilling the RASS-target. The primary outcome was analysed including all days with sedation and after sedation break up to 30 days in an ICU, and separately for the deep sedation, moderate sedation period and the period without sedation. The RASS-target was defined by the leading senior physician at the 7:00 a.m. ward round. The adherence to the RASS-target was subsequently assessed by a RASS scoring at 7:00 p.m. (±1 h) and at 7:00 a.m. at the following day and was either scored positive (target-RASS-score achieved) or negative (target-RASS-score not achieved). We analysed the days with achieved RASS-targets. RASS-target-achieved-days were scored positive only if both target-RASS points in time were adhered to as defined above. The evaluating physicians were aware of the sedation goal and the specific sedative being used but were unaware of the patients’ participation in the study.

### 2.6. Secondary Outcomes

Secondary outcome measures were (i) incidence of delirium, (ii) incidence of ventilator-associated pneumonia (VAP), (iii) neurological functional status at hospital discharge and (iv) in-hospital mortality. Occurrence of delirium was assessed using the Intensive Care Delirium Screening Checklist (ICDSC) at the same timepoints of RASS-assessments. Using the ICDSC, which has also been validated in a German version, symptoms indicative of delirium over the past 24 h are assessed, and the diagnosis is established based on the resulting score [22,23]. The presence, absence, or indeterminability of delirium was documented throughout the entire intensive stay [24]. Incidence of delirium was analysed for each day separately and was scored positive in case of ≥1 positive delirium assessments; otherwise the given day was scored as delirium-negative day. We compared the incidence of delirium, expressed as percentage of patients with one or more days with delirium. To compare the duration of delirium in patients with delirium, the delirium free days were calculated by subtracting the days with delirium from the days without during the moderate- and no-sedation periods. VAP was also scored at the timepoints described above and was defined as the presence of a chest X-ray infiltrate combined with at least two of the following: fever, abnormal leukocyte counts, or purulent bronchial secretions [25]. The incidence of VAP was assessed by comparing the number of patients with VAP to the number of patients without VAP, expressed as a percentage, and the probability of VAP occurrence in the isoflurane- compared to the IV- group was calculated. As described previously, functional neurological outcome at ICU discharge was assessed using the mRS and categorized into favourable versus unfavourable outcome (mRS 0–3 versus mRS 4–6) [26] and also by the NIHSS, by calculation of the difference between NIHSS at discharge to NIHSS at admission [27]. A lower difference shows a clinical functional improvement. Rate of ICU mortality was documented and compared among both sedation groups, stratified for those patients who received withdrawal of life-sustaining treatment (WLST) orders.

### 2.7. Safety Outcomes and Organ Function

We a priori defined several safety measures, i.e., (i) if norepinephrine dosages exceeding 0.5 µg kg^−1^ min^−1^ are given the sedation with isoflurane will be stopped [28] and (ii) the occurrence of transient pupillary dilatation during the sedation periods, as described previously to eventually occur while using volatile anaesthetics, would also stop the sedation with isoflurane [29]. During the study period, various laboratory parameters were collected to monitor organ function and compare outcomes between both groups. Renal function was assessed through daily measurements of creatinine and urea. Liver function was monitored by measuring gamma-glutamyltransferase (GGT), aspartate aminotransferase (AST) and bilirubin. The hematopoietic system was evaluated through daily measurements of leukocyte count, haemoglobin and platelet count, while ventilation status was monitored via arterial partial-pressure of carbon dioxide (paCO_2_) levels.

### 2.8. Statistical Analysis

All statistical analyses were performed using RStudio (v2024.09.0, Posit Software, Boston, MA, USA) and R version 4.4.0 (R Project for Statistical Computing). A 2-sided α of 0.05 was used to connote significance for all statistical tests. The normality of the data distribution was tested using the Shapiro–Wilk test. Student’s *t* test for independent samples, Fisher’s exact test, the Mann–Whitney U test (M-W-U), and Pearson’s chi-squared test were used to compare the two sedation groups, each as appropriate. Data are presented as mean values ± standard deviations (SDs), median values with the 25th to 75th interquartile range [Q_0.25_–Q_0.75_], or total counts with percentages, as appropriate. The association between isoflurane vs. IV sedation and the predefined outcome parameter was quantified using the Risk Ratio (RR) calculated with the Wald method to provide a 95% confidence interval, assessing precision in the estimate. Analyses were conducted using the “epitools” package in R [30]. Statistical significance was determined using Fisher’s exact test.

A linear mixed-effects model was used to assess differences in time-dependent parameters between the IV and isoflurane groups over time. The model included the parameter under investigation as the dependent variable, with group (isoflurane vs. IV), time (days) and their interaction as fixed effects, and a random intercept for each model to account for baseline variability. The model was fit using Restricted Maximum Likelihood (REML) with the “lme4” package [31]. Since this is an observational, non-randomized study, a propensity score analysis followed by matching was conducted despite highly comparable physiological parameters in both groups. The analysis was performed using propensity score matching with the R package “MatchIt”. This applies a nearest-neighbour matching algorithm with a 1:1 ratio and a caliper 0.1 using logistic regression to calculate the propensity scores. The treatment group was matched to a control group based on the following covariates: age, side of stroke, initial SOFA score, sex, initial NIHSS score and BMI. The study analysis was then repeated with the reduced, matched cohort as previously described.

## 3. Results

During the 12-month screening period, a total of 89 patients were considered eligible for participation based on the inclusion and exclusion criteria. Of these, 10 patients were excluded because of declining consent, where 79 patients remained for analysis. Comparing the excluded with the included patients did not reveal any relevant imbalances or heterogeneity (Supplementary Table S1). Of all enrolled patients, 36/79 (46%) were allocated to the isoflurane-group and 43/79 (54%) to the IV-group (Supplementary Figure S2). The baseline characteristics were balanced without significant differences in demographic, clinical, laboratory and treatment aspects among both groups (Table 1). To verify an unbiased proceeding with the entire cohort, propensity score matching demonstrated relatively small quartile differences between both groups (Supplementary Table S2 and Supplementary Figure S3) without differences in analyses of primary and secondary outcomes.

### 3.1. Primary Outcome

**Figure 1:**
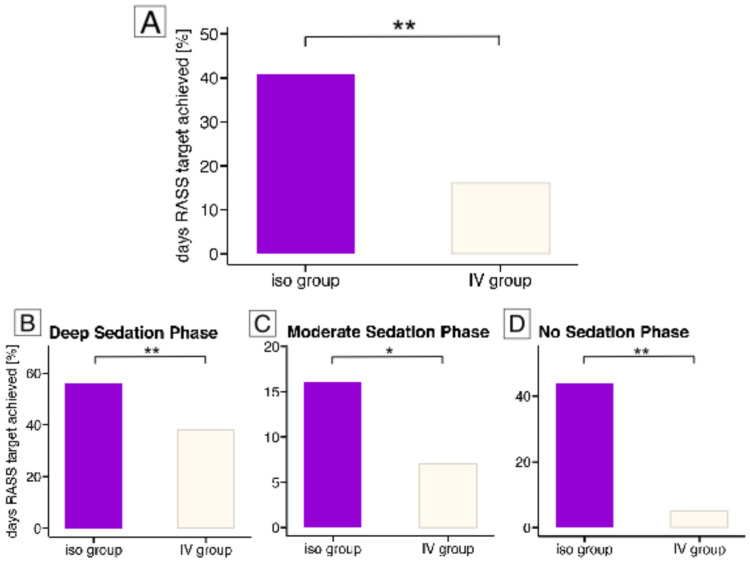
Achieved sedation targets. This figure shows the primary outcome results. Panel (**A**) displays the percentage of days across the entire cohort on which the sedation target was met at both time points. Panels (**B**–**D**) show the days with target achievement in the three distinct sedation phases: (**B**) deep sedation, (**C**) moderate sedation and (**D**) no sedation. Violet bars represent the isoflurane group (iso group), and white bars represent the IV group. Asterisks (*) indicate significant differences between groups, with * for *p* < 0.05 and ** for *p* < 0.001.

Overall, there were 941 days with RASS scores available at both time points over a mean ICU stay of 11 [7–19] days. Of these, 262 days were scored RASS-target achieved and 679 RASS-target not achieved. Compared to IV-grouped patients, who revealed 80/497 (16%) days on RASS-target levels, those of the isoflurane-group showed with 182/444 (41%) significantly more days with reached target level (RR 1.42; 95%-CI 1.31 to 1.55, *p* < 0.001; Figure 1). A detailed analysis of the two RASS assessments, differentiated by morning and evening measurements, is provided in the Supplementary Materials.

Focusing on individual phases of sedation depth, there were a total of 344 days with a deep sedation target (RASS ≤ −4), 304 days with a moderate sedation target (RASS −3–−1), and 372 days with a no-sedation target (RASS 0). There were significant differences in favour of the isoflurane-group compared to the IV-group in achieving all of the various sedation targets, specifically with (i) the deep sedation target (165/340 (48%) days of all patients with 105/186 (56%) days in the isoflurane- and 60/157 (38%) days in the IV-group (RR 1.5; 95%-CI: 1.2–1.9; *p* = 0.001), Figure 1B), (ii) the moderate sedation target (33/286 (12%) of all patients with 22/134 (16%) in the isoflurane- and 11/155 (7%) in the IV-group (RR 1.7, 95%-CI: 1.0–2.7; *p* = 0.02), Figure 1C) and (iii) the no-sedation target (64/309 (21%) of the days in the entire cohort with 55/124 (44%) in the isoflurane- and 9/185 (5%) days in the IV-group (RR 5.1; 95%-CI: 2.8–9.4; *p* < 0.001), Figure 1D).

### 3.2. Secondary Outcomes

**Figure 2:**
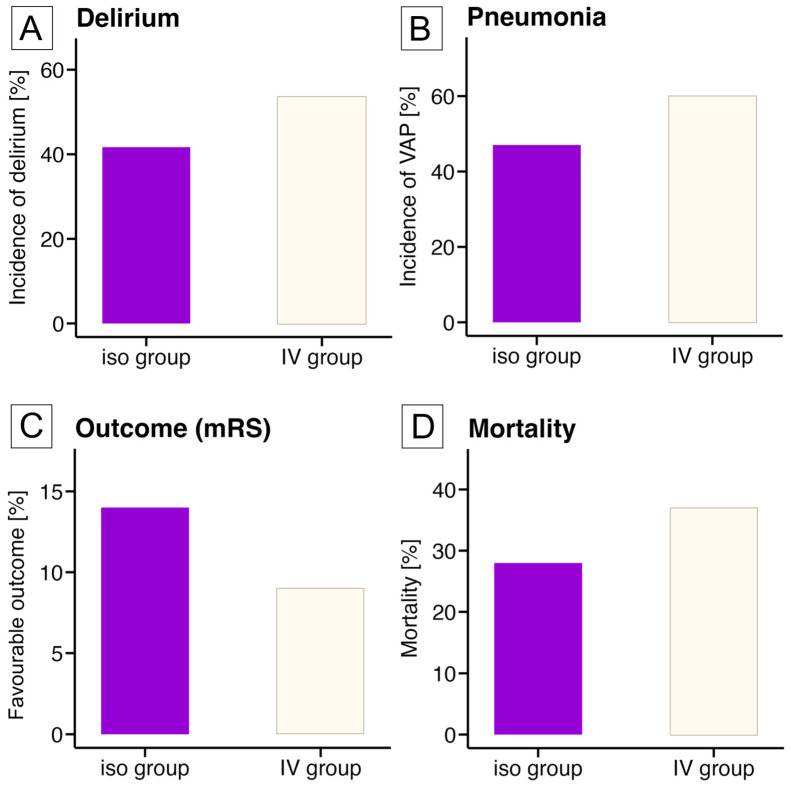
Secondary outcomes. The figure presents the predefined secondary outcomes. The left upper panel (**A**) shows the incidence of delirium in percent, assessed via the Intensive Care Delirium Screening Checklist. The right upper panel (**B**) indicates the percentage of patients with ventilator-associated pneumonia (VAP). The left lower panel (**C**) displays the percentage of patients with a favourable outcome (RASS 0-3 at ICU discharge), while the right lower panel (**D**) shows the mortality rate. Violet bars represent the isoflurane group (iso group), and white bars represent the IV group.

The secondary outcomes are displayed in Figure 2. Deliria were observed in 38/79 (48%) of all patients with a non-significant risk reduction in the isoflurane-group compared to the IV-group (isoflurane: 15/36 (42%) vs. IV: 23/43 (54%); RR = 0.81; 95%-CI: 0.54–1.21; *p* = 0.37, Figure 2A). Ventilator-associated pneumonia occurred in 43/79 (54%) of all patients with a non-significant risk reduction in the isoflurane-group compared to the IV-group (isoflurane: 17/36 (47%) vs. IV: 26/43 (60%), RR = 0.78; 95%-CI: 0.51–1.19; *p* = 0.26, Figure 2B). The proportion of patients with a favourable vs. unfavourable functional outcome was comparable among both groups (mRS-0-3: isoflurane: 5/36 (14%) vs. IV: 4/43 (9%); RR = 1.25; 95%-CI: 0.58–2.67; *p* = 0.72, Figure 2C). Mortality rates were not significantly different between groups (isoflurane: 10/36 (28%) vs. IV: 16/43 (37%); RR of 0.83; 95%-CI: 0.55–1.24; *p* = 0.47, Figure 2D) and were mainly driven by withdrawal of life-sustaining treatment during the course of disease. 

### 3.3. Safety Outcomes and Organ Function

**Figure 3:**
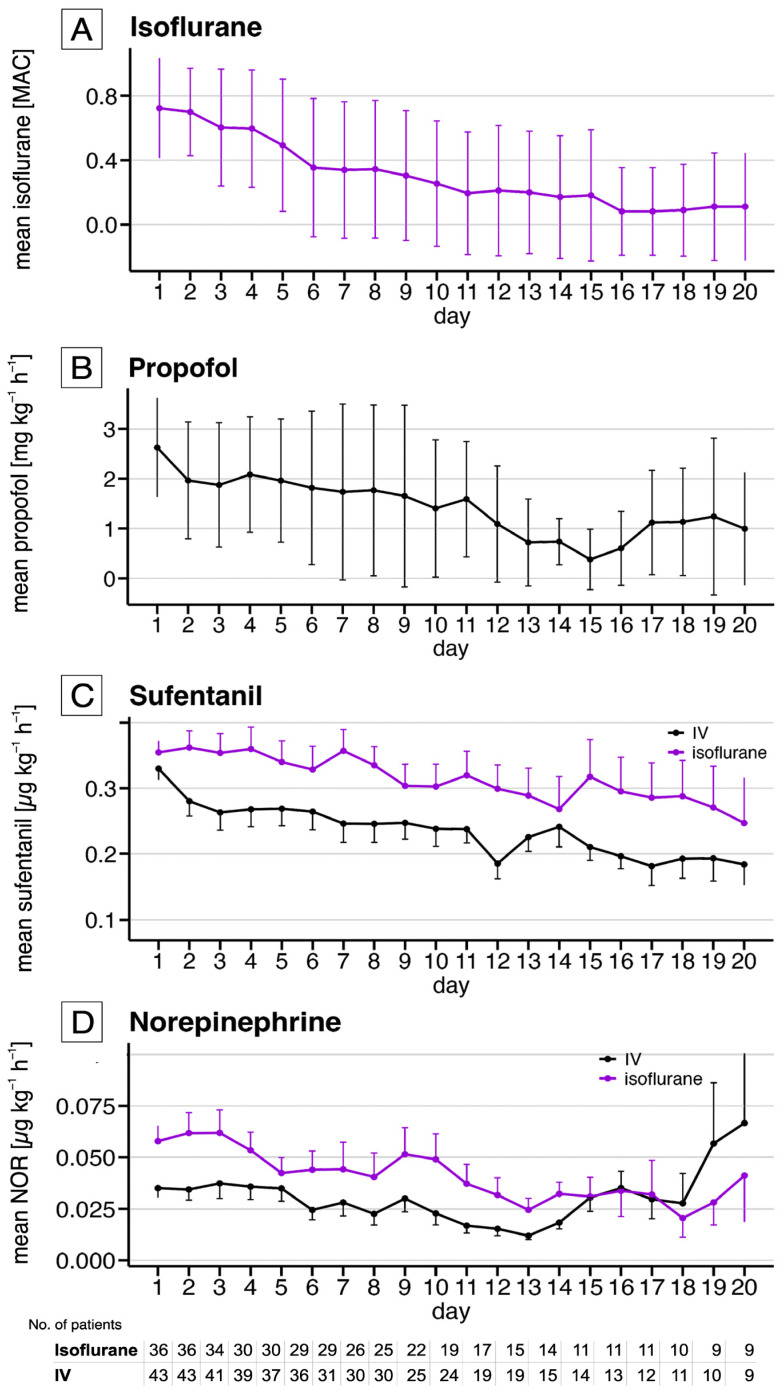
Mean doses of sedatives, analgetic and norepinephrine. The upper graph (**A**) displays the daily average mean alveolar concentration (MAC) of isoflurane for the isoflurane group only, while the middle graph (**B**) presents the daily average doses of propofol for the IV group only. The lower graphs (**C**,**D**) combines both groups, showing the daily mean dosing of sufentanil (**C**) and the daily mean doses of norepinephrine (**D**), with the isoflurane group in violet and the IV group in black. For each group, daily means and standard deviations across all patients are presented for the first 20 days, as patient numbers beyond this point were insufficient for reliable analysis.

Figure 3 provides an overview of the administered dosages of sedatives, analgesics and vasopressors. The administered doses of sufentanil were higher in the isoflurane- compared to the IV-group (Figure 3C; *p* < 0.001; Supplementary Table S3). The median norepinephrine doses were 0.031 ± 0.01 µg kg^−1^ min^−1^ (isoflurane: 0.033 ± 0.01 µg kg^−1^ min^−1^ vs. IV: 0.028 ± 0.01 µg kg^−1^ min^−1^; *p* = 0.001, Figure 3D). The observed differences of sufentanil and norepinephrine in unfavourable outcomes of the isoflurane group were clinically not relevant and the predefined thresholds were not reached.

Unexpected mydriatic changes occurred in one patient of each group. In the isoflurane group mydriasis lasted for 2 days and in the IV group for 4 days, all without changes upon CT imaging.

The renal function was monitored by creatinine and urea and showed no differences between the groups (Supplementary Figure S4). Differences among laboratory values of liver function and the hematopoietic system between groups were not statistically significant (Supplementary Figures S5 and S6), as were the differences in paCO_2_ levels (Supplementary Figure S7).

## 4. Discussion

To the best of our knowledge, this is the first prospective systematic analysis of associations of isoflurane sedation with clinical outcomes in critically ill patients with stroke. In essence, here, we (i) demonstrate that isoflurane sedation is feasible in daily routine without relevant safety concerns, (ii) provide evidence that isoflurane sedation is superior to propofol sedation in achieving the sedation levels targeted for each day during hospital stay, (iii) verify that isoflurane-sedated patients exhibited faster recovery from deep sedation states and (iv) show promising trends towards reduced incidence of delirium and ventilator-associated pneumonia, which serve as a robust basis for effect size estimations in subsequent studies on the significance of inhaled sedation in stroke patient care. 

Three aspects emerge from the data. First, given that sedation in intensive care requires a precise balancing, volatile aesthetic drugs should be considered more broadly for long-term sedation in (neuro-)critical care. While minimal sedation is crucial for neurological monitoring, patient interaction, and early rehabilitation, a deep sedation plays a pivotal role in the acute phase of acute brain injury with significant mass effect, mainly driven by neuroprotection due to reduced cerebral metabolism [4]. Hence, a facilitated handling to titrate sedation targets more efficiently, as demonstrated here for critically ill stroke patients, certainly appears valuable, specifically the aspect of a more precise awakening control. Similar data of a superior efficacy of isoflurane over propofol in achieving specific sedation targets and its feasibility and advantage on awakening times have been reported previously [9,13,32]. While these studies included no or only limited neurological cases, our findings extend our knowledge and contribute to a better understanding of isoflurane in neurocritical care. Of note, in this regard, is that leading specialized centres have already started implementing inhaled sedatives in their clinical practice and recognizing their potential [29]. However, to further promote this trend, more studies on volatile anaesthetics appear warranted.

Second, with respect to the presented promising data on secondary outcomes, notably rates of delirium and VAP, there have been various studies for non-neurological patients [33,34]. Reduced rates of de-synchronization between ventilation triggers of the patients’ versus that of mechanical ventilation devices have been proposed as a mechanism for fewer deliria in intensive care and post-cardiac arrest patients [34]. Further, specific neuroprotective effects with reducing hippocampal neuronal apoptosis have been attributed to isoflurane in animal studies [35]. We detected similar findings for VAP, which represents a severe post-stroke complication that substantially increases morbidity and mortality [36]. As suggested here, isoflurane sedation in stroke patients is associated with fewer rates of VAP, potentially attributable to enhanced spontaneous breathing patterns, decreased atelectasis formation and improved secretion clearance, all of which may accelerate recovery [33]. Yet, these beneficial associations of volatile anaesthetics with reduced incidence of deliria and VAP were unexplored in stroke patients, and our findings certainly appear relevant, requiring further analysis in future studies. For the latter, data of this study may serve for effect size and power calculations and as a basis for confirmatory studies with clinical outcomes.

Third, and at a first glace most important, the use of isoflurane was feasibly and safe in neurocritical care patients. Given that isoflurane leads to vasodilatation, necessitating administration of vasopressors, previous data with neurological patients reported a greater need for vasopressors [12]. These findings, however, were not observed in patients with acute respiratory distress syndrome (ARDS) [32]. Here, we verified that patients with isoflurane required more norepinephrine; however, the additional requirement was minimal, which is consistent with findings in subarachnoid haemorrhage [37], and clinically not relevant. Further concerns to widely use isoflurane include unexpected mydriasis [29,38]. However, in our study, mydriasis was rare and evenly distributed across sedation groups, which is a finding in line with recent meta-analyses that do not list mydriasis as a common adverse effect [39,40]. Practitioners should remain vigilant for pupillary problems but should generally not refrain from isoflurane use. Finally, there were no signals of harm of isoflurane compared to propofol with regard to opioid use or side effects on organ functions. These findings should promote a broader use and further clinical studies using volatile anaesthetics in patients with severe neurological disease.

Despite certain strengths of our study, notably enrolling neurocritical care patients previously under-represented in existing studies and sound analysis of outcomes including safety aspects, there are various limitations. The main shortcomings include lack of patient-based randomization and blinding of clinical staff with respect to treatment allocation. This method could inadvertently correlate with unmeasured factors which might affect our results. Although baseline characteristics were largely comparable between groups, we cannot completely exclude residual confounding factors. Further, we did not perform additional neuro-monitoring, which precludes direct conclusions regarding associations with intracranial pressure that, however, appear unproblematic [41]. Finally, though baseline parameters were comparable among both groups, there is residual uncertainty and bias by indication why non-assessed parameters, and differences between both centres may have influenced primary and secondary outcomes.

## 5. Conclusions

The results of this prospective observational pilot study suggest that isoflurane sedation offers valuable benefits for invasively ventilated stroke patients. Specifically, it facilitates faster awakening when transitioning from deep to moderate sedation. Further, isoflurane shows positive associations with secondary outcomes, notably delirium and ventilator-associated pneumonia, without any side effects or safety concerns. In light of the demonstrated advantages of isoflurane, the data provided here may serve as a robust foundation and effect size estimates for future confirmatory studies evaluating volatile anaesthetics in stroke patients.

## Figures and Tables

**Table 1:** Patient characteristics and group comparison.

	Isoflurane	Propofol	Significance
Number [*n*]	36	43	
Patients’ characteristics:			
Age [years]	71 [64–80]	73 [62–82]	0.98 *
Female sex [*n* (%)]	13 (36%)	18 (42%)	0.77 ^+^
BMI [kg m^−2^]	27 [25–29]	28 [25–29]	0.63 *
Pre-mRS	1 [0–2]	0 [0,1]	0.14 *
SOFA at admission	10 [9–11]	10 [9–11]	0.13 *
NIHSS at admission	17 [13–20]	17 [12–20]	0.91 *
Stroke characteristics:			
Affected side:			0.22 ^+^
Right [*n* (%)]	21 (58%)	17 (40%)	
Left [*n* (%)]	11 (31%)	21 (49%)	
Multilocular [*n* (%)]	4 (11%)	5 (12%)	
ICH [*n* (%)]	4 (11%)	2 (5%)	0.42 ^+^
lobar [*n*]	1	1	
Basal ganglia [*n*]	3	1	
Volume [mL]	33 ± 15	30 ± 0	0.68 ^#^
AIS [*n* (%)]	32 (89%)	41 (95%)	0.51 ^+^
M1 [*n* (%AIS)]	21 (66%)	28 (68%)	0.91 ^+^
M2 [*n* (%AIS)]	5 (16%)	5 (12%)	
BA [*n* (%AIS)]	6 (19%)	8 (20%)	
Extension ^§^ [%]	63 [50–75]	50 [50–75]	0.23 ^+^
IVT [*n* (%AIS)]	14 (44%)	12 (30%)	0.43 ^+^
MT [*n* (%AIS)]	24 (75%)	33 (80%)	0.45 ^+^
DC [*n* (%AIS)]	3 (9%)	3 (7%)	1
Laboratory at admission:			
Leucocyte count [10^9^ L^−1^]	11.1 ± 4	11.5 ± 4	0.98 ^#^
Haemoglobin [g L^−1^]	128 ± 24	126 ± 23	0.65 ^#^
Platelet count [10^9^ L^−1^]	233 [202–269]	223 [183–275]	0.66 *
PT [Int. Ratio]	1.0 [1.0–1.1]	1.1 [1.0–1.3]	0.01 *
GGT [U L^−1^]	29 [22–45]	27 [20–46]	0.60 *
AST [U L^−1^]	25 [22–31]	22 [18–34]	0.60 *
Bilirubin [µmol L^−1^]	8.6 [8.1–12.4]	10.3 [6.8–15.4]	0.99 *
Creatinine [µmol L^−1^]	79.6 [62–100]	79.6 [62–97]	0.36 *
Urea [mmol L^−1^]	5.9 [4.4–7.5]	6.0 [5.2–8.0]	0.60 *
Treatment characteristics:			
Length of ICU stay [days]	11 [7–19]	11 [6–18]	0.92 *
Duration of ventilation [h]	266 [183–469]	279 [174–454]	0.99 *

The table shows patient’ characteristics, types of strokes, laboratory parameters at admission and treatment characteristics in the two groups. After a test of data distribution (not shown) the parameters were tested for significant differences using a *t*-test ^#^ for normally distributed data, χ^2^ test ^+^ for dichotomous data and Mann–Whitney U test * for non-normally distributed data. IV: intravenous sedation group; BMI: body mass index; Pre-mRS: modified Rankin scale before stroke; SOFA: sequential organ failure assessment; NIHSS: national institute of health stroke scale; ICH: intracranial haemorrhage; AIS: acute ischemic stroke; M1: affected vessel mean cerebral artery segment 1; M2: affected vessel mean cerebral artery segment 2; BA: affected vessel basilar artery; IVT: intravenous thrombolysis; MT: mechanical thrombectomy; DC: decompressive craniectomy; PT: prothrombine time; GGT: gamma-glutamyltransferase; AST: aspartate aminotransferase; and ICU: intensive care unit. Parameters are expressed as numbers and percentage [*n* (%)], means ± standard deviations, or medians [Q_0.25_–Q_0.75_]. ^§^ extent of the infarction proportional to the vascular territory.

## Data Availability

The datasets generated and analysed during the current study are not publicly available due to pseudonymization but are available from the corresponding author on reasonable request.

## Supplementary Materials

The following supporting information can be downloaded at: https://www.mdpi.com/article/10.3390/jcm14051594/s1: Supplementary Figure S1: Standard operating procedure for sedation; Supplementary Figure S2: Screening and allocation flowchart; Supplementary Figure S3: Propensity score matching; Supplementary Figure S4: Time course of renal function parameters; Supplementary Figure S5: Time course of the liver function parameters; Supplementary Figure S6: Time course of the hematopoietic system parameters; Supplementary Figure S7: Time course of the paCO_2_; Supplementary Table S1: Patient characteristics and comparison with excluded patients; Supplementary Table S2: Results of the propensity score matching; Supplementary Table S3: Mean doses of sedatives and analgesics.

## Abbreviations

The following abbreviations are used in this manuscript:

AIS	acute ischemic stroke
ARDS	acute respiratory distress syndrome
AST	aspartate aminotransferase
BMI	body mass index
CSHT	context sensitive half-time
GGT	gamma-glutamyltransferase
ICDSC	Intensive Care Delirium Screening Checklist
ICH	intracerebral haemorrhage
ICU	intensive care unit
MAC	minimum alveolar concentration
MCA	middle cerebral artery
mRS	modified Rankin scale
NIHSS	national institute of health stroke scale
paCO2	arterial partial-pressure of carbon dioxide
RASS	Richmond-Agitation-Sedation-Scale
rt-PA	tissue-type plasminogen activator
SOFA	sequential organ failure assessment
VAP	ventilator-associated pneumonia
WLST	withdrawal of life-sustaining treatment
