# Supplementary material for: Isoflurane vs. Propofol Sedation in Patients with Severe Stroke: A Clinical Proof-of-Concept-Study
Source: J Clin Med. 2025 Feb 26;14(5):1594. doi: 10.3390/jcm14051594 (PMC11901015; doi:10.3390/jcm14051594)
Supplement: Supplementary file 1 [file jcm-14-01594-s001.zip › jcm-3465442-supplementary.pdf]

Isoflurane vs. Propofol Sedation in Patients with Severe Stroke:

A Clinical Proof-of-Concept-Study

Supplement

Content

Supplementary Figure S1: Standard operating procedure for sedation........................ 2

Supplementary Table S1: Patient characteristics and comparison with excluded patients.. 3

Supplementary Figure S2: Screening and allocation flowchart.................................... 5

Supplementary Table S2: Results of the propensity score matching........................... 6

Supplementary Figure S3: Propensity score matching 7

Supplementary detailed analysis of the two daily RASS assessments......................... 8

Supplementary Table S3: Mean doses of sedatives and analgesics 9

Supplementary detailed analysis of organ function over time.................................... 10

Renal function.. 10

Supplementary Figure S4: Time course of renal function parameters........................ 10

Liver function.. 11

Supplementary Figure S5: Time course of the liver function parameters 11

Hematopoietic system.. 13

Supplementary Figure S6: Time course of the hematopoietic system parameters...... 13

Carbon Dioxid 15

Supplementary Figure S7: Time course of the $paCO_2$... 15

Figure S1. Standard operating procedure sedation.

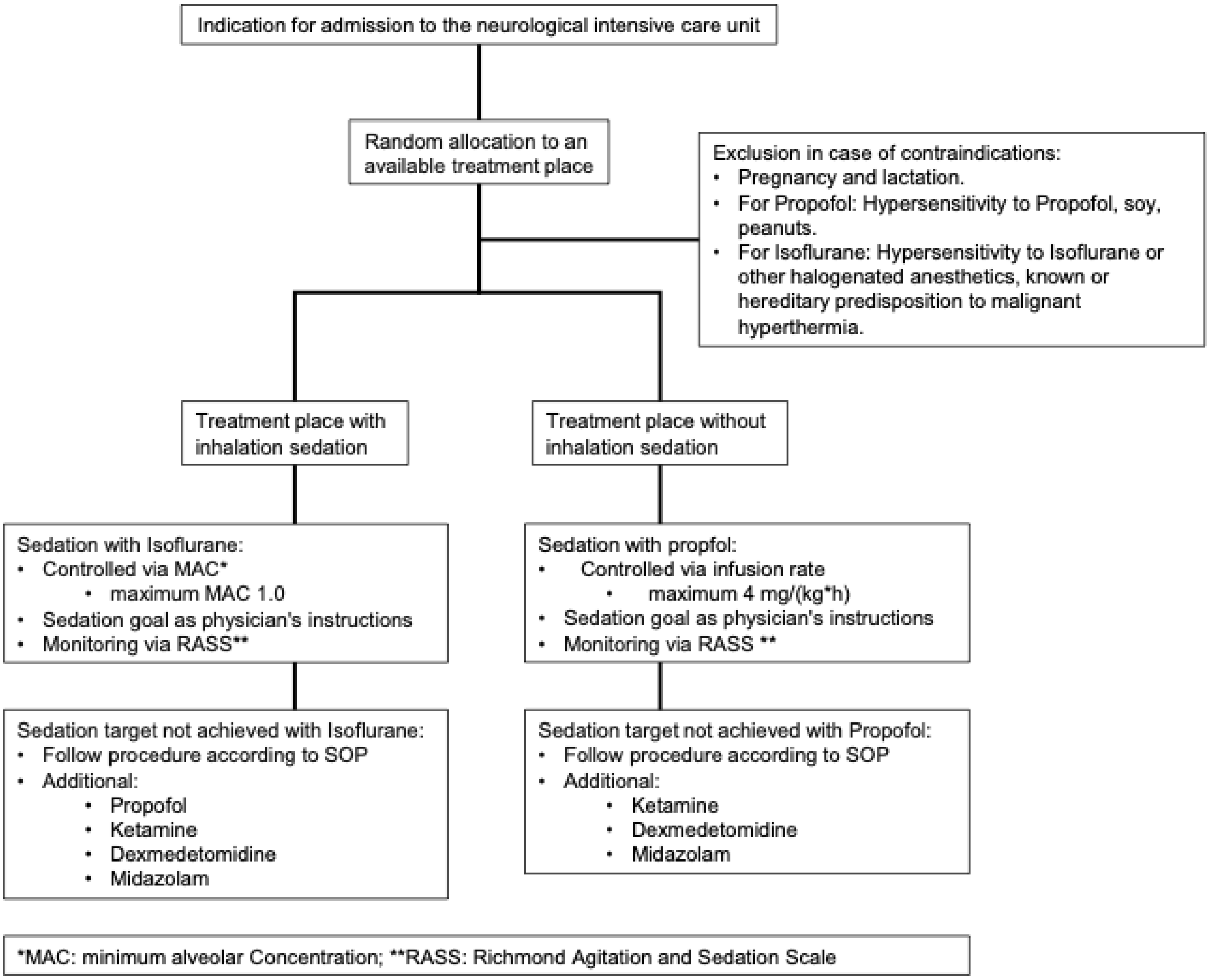

This figure illustrates the standard operating procedure (SOP) that, independently of the observational study presented here, outlines how patients with severe stroke are assigned to a bed and how sedation is managed, depending on whether inhalation sedation is available at the assigned bed.

Table S1. Patient characteristics and comparison with excluded patients.

	Study population	Excluded patients	significance
**Number [n]**	79	10	
**Patients' characteristics:**			
**Age [years]**	71 [63-81]	78 [70-88]	0.10*
**Female sex [n, (%)]**	31 (39%)	8 (80%)	0.04+
**BMI [kg m^{-2}]**	27 [25-29]	25 [24-26]	0.04*
**Pre-mRS**	0 [0-2]	1 [1-2]	0.22*
**SOFA at admission**	10 [9-11]	7 [7-8]	<0.05*
**NIHSS at admission**	17 [12-20]	19 [13-30]	0.28*
**Stroke characteristics:**			
**Affected side:**			0.85+
**Right [n (%)]**	38 (48%)	4 (40%)	
**Left [n (%)]**	32 (41%)	5 (50%)	
**Multilocular [n (%)]**	9 (11%)	1 (10%)	
**ICH [n (%)]**	6 (8%)	0	
**lobar [n]**	2		
**Basal ganglia [n]**	4		
**Volume [ml]**	33 ± 15		
**AIS [n (%)]**	73 (92%)	10 (100%)	
**M1 [n (%AIS)]**	49 (67%)	6 (60%)	0.37+
**M2 [n (%AIS)]**	10 (14%)	3 (30%)	
**BA [n (%AIS)]**	14 (19%)	1 (10%)	
**Extension§ [%]**	50 [50-75]	75 [50-75]	0.44+
**IVT [n(%AIS)]**	26 (36%)	4 (40%)	0.93+
**MT [n(%AIS)]**	57 (78%)	9 (90%)	0.41+
**DC [n(%AIS)]**	6 (8%)	0	
**Laboratory at admission:**			
**Leucocyte count [10^9 l^{-1}]**	11.1 ± 4	10.8 ± 3	0.79#
**Haemoglobin [g l^{-1}]**	127 ± 24	120 ± 22	0.39#
**Platelet count [10^9 l^{-1}]**	231 [184-275]	251 [196-344]	0.39*
**PT [Int. Ratio]**	1.1 [1.0-1.1]	1.0 [1.0-1.2]	0.58*
**GGT [U l^{-1}]**	28 [20-46]	26 [15-38]	0.46*
**AST [U l^{-1}]**	24 [19-33]	24 [22-34]	0.58*
**Bilirubin [µmol l^{-1}]**	8.6 [6.8-14.5]	8.6 [6.8-11.5]	0.66*
**Creatinine [µmol l^{-1}]**	79.6 [62-97]	70.7 [62-86]	0.65*
**Urea [mmol l^{-1}]**	6.0 [5.0-7.9]	5.9 [5.4-9.3]	0.59*
**Treatment characteristics**			
**Isoflurane sedation [n (%)]**	36 (46%	4 (40%)	
**Length of ICU stay [days]**	11 [7-18]	13 [8-24]	0.60*
**Duration of ventilation [hours]**	273 [177-459]	176 [125-270]	0.12*

The table shows patients' characteristics, types of strokes, laboratory parameters at admission and treatment characteristics in the whole study population and in the population of excluded patients. After a test of data distribution (not shown) the parameters were tested of significant differences using a t-test # for normally distributed data, χ^2 test + for dichotomous

data and *Mann–Whitney U test* [*] *for non-normally distributed data. IV: intravenous sedation group; BMI: body mass index; Pre-mRS: modified Rankin scale before stroke; SOFA: sequential organ failure assessment; NIHSS: national institute of health stroke scale; ICH: intracranial haemorrhage; AIS: acute ischemic stroke; M1: affected vessel mean cerebral artery segment 1; M2: affected vessel mean cerebral artery segment 2; BA: affected vessel basilar artery; IVT; intravenous thrombolysis; MT: mechanical thrombectomy; DC: decompressive craniectomy; PT: prothrombine time; GGT: Gamma-Glutamyltransferase; AST: aspartate aminotransferase; and ICU: intensive care unit.*
Parameters are expressed as numbers and percentage [n (%)], means ± standard deviations or medians [Q0.25-Q0.75]. [§] *Extent of the infarction proportional to the vascular territory*

Figure S2. Screening and allocation flowchart.

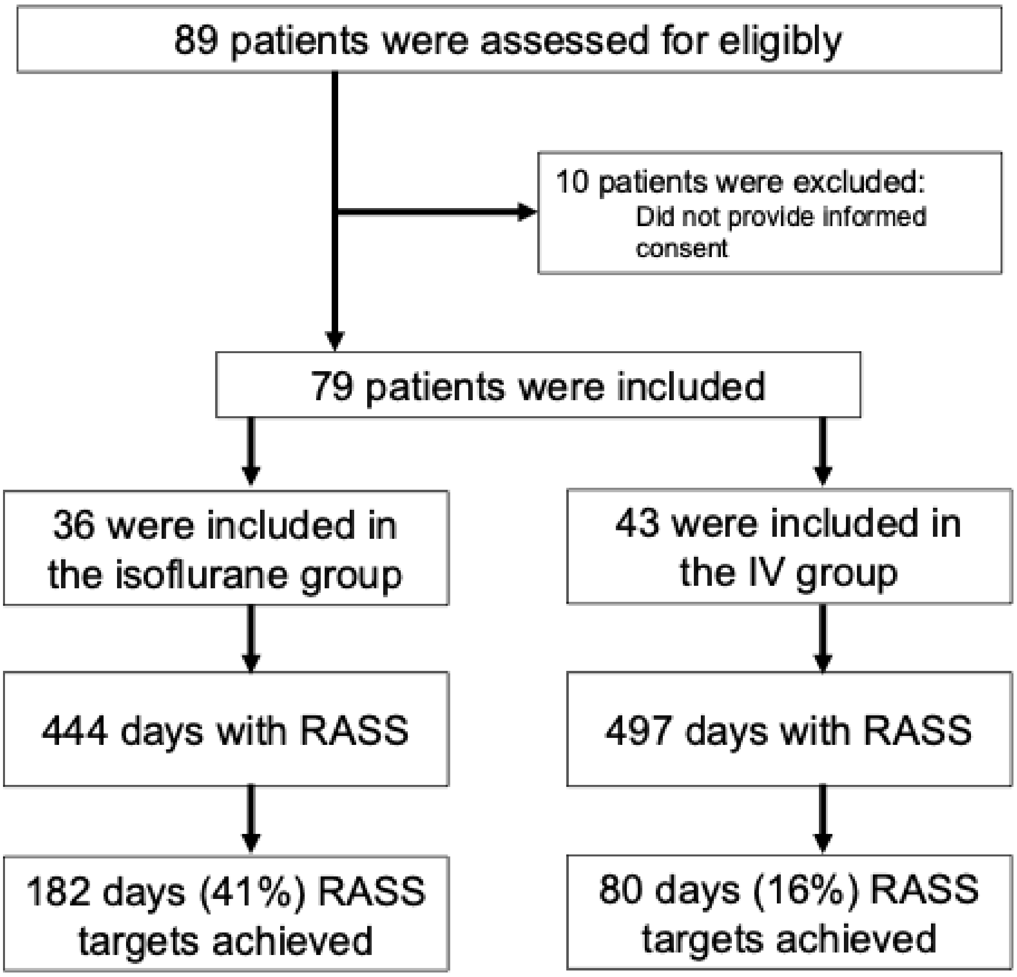

This flowchart illustrates patient screening, allocation to the two treatment groups, the total number of days with complete RASS evaluations and the resulting percentage of achieved RASS targets.

Table S2. Results of the propensity score matching.

A	Means Treated	Means Control	Std. Mean Diff.	Var. Ratio	eCDF Mean	eCDF Max	Std. Pair Dist.
distance	0.533	0.391	0.725	1.465	0.211	0.305	NA
age	71.306	70.558	0.067	0.827	0.032	0.072	NA
stroke side	1.528	1.721	-0.277	1.092	0.064	0.188	NA
SOFA	10.250	9.977	0.204	0.731	0.072	0.187	NA
sex	0.361	0.419	-0.120	NA	0.057	0.057	NA
stroke entity	1.889	1.953	-0.203	2.237	0.032	0.065	NA
performed thrombolysis	0.389	0.279	0.225	NA	0.110	0.110	NA
performed thrombectomy	0.667	0.767	-0.214	NA	0.101	0.101	NA
NIHSS	16.556	16.209	0.053	0.661	0.049	0.130	NA
pre mRS	1.306	0.791	0.350	1.709	0.086	0.212	NA
BMI	27.679	28.118	-0.075	1.148	0.035	0.118	NA
ICH location	0.139	0.047	0.218	3.968	0.031	0.065	NA
AIS expansion	0.576	0.570	0.024	1.630	0.049	0.096	NA

B	Means Treated	Means Control	Std. Mean Diff.	Var. Ratio	eCDF Mean	eCDF Max	Std. Pair Dist.
distance	0.533	0.432	0.517	1.852	0.141	0.278	0.517
age	71.306	71.861	-0.049	0.928	0.035	0.139	1.222
stroke side	1.528	1.694	-0.239	1.085	0.056	0.167	0.878
SOFA	10.250	10.111	0.104	0.671	0.069	0.139	1.265
sex	0.361	0.389	-0.058	NA	0.028	0.028	0.867
stroke entity	1.889	1.944	-0.174	1.882	0.028	0.056	0.174
performed thrombolysis	0.389	0.333	0.114	NA	0.056	0.056	0.684
performed thrombectomy	0.667	0.722	-0.118	NA	0.056	0.056	0.589
NIHSS	16.556	16.639	-0.013	0.704	0.035	0.111	1.012
pre mRS	1.306	0.917	0.265	1.552	0.065	0.167	0.718
BMI	27.679	27.898	-0.038	1.132	0.032	0.111	1.025
ICH location	0.139	0.056	0.196	3.338	0.028	0.056	0.196
AIS expansion	0.576	0.583	-0.025	1.388	0.028	0.056	0.586

Table A presents the summary of the propensity score analysis of the whole cohort and table B shows the summary after matching when seven patients of the IV-group were excluded. BMI: body mass index; Pre-mRS: modified Rankin scale before stroke; SOFA: sequential organ failure assessment; NIHSS: national institute of health stroke scale; ICH: intracranial haemorrhage; and AIS: acute ischemic stroke.

Figure S3. Propensity score matching.

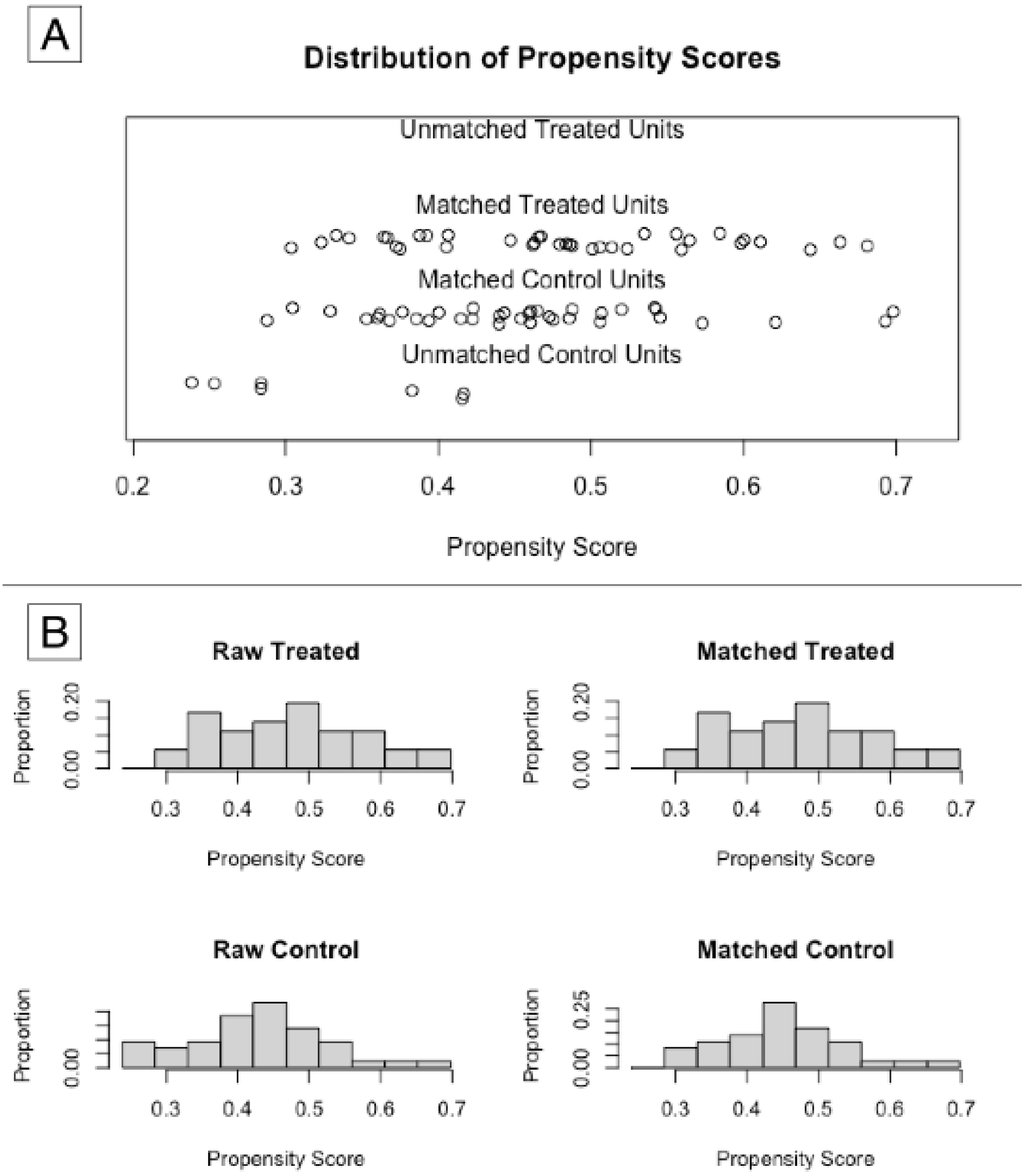

The figure shows the results of the propensity score matching.

Supplementary detailed analysis of the two daily RASS assessments

A total of 1958 RASS assessments were performed following the RASS target definition visit. Of these, 1017 RASS scores were recorded during the evening visit (12 hours post-target definition), and 941 during the morning visit (24 hours post-target definition). After 12 hours, 348 out of 1017 RASS scores (34%) reached the targeted sedation depth. In the isoflurane-group, the target RASS score was achieved in 231 out of 478 assessments (48%) during the evening visit, compared to 117 out of 539 assessments (22%) in the IV-group, corresponding to a RR of 1.52 (95% CI: 1.37–1.67, $p < 0.001$). After 24 hours, 317 out of 941 RASS Scores (34%) reached the targeted sedation depth. In the isoflurane-group, the target RASS score was achieved in 210 out of 444 assessments (47%) during the visit on the following morning, compared to 107 out of 497 assessments (22%) in the IV-group, corresponding to a RR of 1.49 (95% CI: 1.35–1.65, $p<0.001$).

Table S3. Mean doses of sedatives and analgesics.

	**isoflurane**			**IV**	
day	MAC	Sufentanil [µg kg^{-1} h^{-1}]		Propofol [mg kg^{-1} h^{-1}]	Sufentanil [µg kg^{-1} h^{-1}]
**1**	0.7 ± 0.3	0.35 ± 0.10		2.6 ± 1.0	0.33 ± 0.11
**2**	0.7 ± 0.3	0.36 ± 0.15		2.0 ± 1.2	0.28 ± 0.15
**3**	0.6 ± 0.4	0.35 ± 0.17		1.9 ± 1.2	0.26 ± 0.17
**4**	0.6 ± 0.4	0.36 ± 0.18		2.1 ± 1.2	0.27 ± 0.16
**5**	0.5 ± 0.4	0.34 ± 0.18		2.0 ± 1.2	0.27 ± 0.16
**6**	0.4 ± 0.4	0.33 ± 0.19		1.8 ± 1.5	0.26 ± 0.17
**7**	0.3 ± 0.4	0.36 ± 0.18		1.7 ± 1.8	0.25 ± 0.16
**8**	0.3 ± 0.4	0.34 ± 0.15		1.8 ± 1.7	0.25 ± 0.16
**9**	0.3 ± 0.4	0.30 ± 0.16		1.7 ± 1.8	0.25 ± 0.14
**10**	0.3 ± 0.4	0.30 ± 0.16		1.4 ± 1.4	0.24 ± 0.13
**11**	0.2 ± 0.4	0.32 ± 0.16		1.6 ± 1.2	0.24 ± 0.10
**12**	0.2 ± 0.4	0.30 ± 0.15		1.1 ± 1.2	0.19 ± 0.10
**13**	0.2 ± 0.4	0.29 ± 0.16		0.7 ± 0.9	0.23 ± 0.10
**14**	0.2 ± 0.4	0.27 ± 0.19		0.7 ± 0.5	0.24 ± 0.12
**15**	0.2 ± 0.4	0.32 ± 0.19		0.4 ± 0.6	0.21 ± 0.07
**16**	0.1 ± 0.3	0.30 ± 0.17		0.6 ± 0.7	0.20 ± 0.07
**17**	0.1 ± 0.3	0.29 ± 0.18		1.1 ± 1.0	0.18 ± 0.10
**18**	0.1 ± 0.3	0.29 ± 0.17		1.1 ± 1.1	0.19 ± 0.10
**19**	0.1 ± 0.3	0.27 ± 0.19		1.2 ± 1.6	0.19 ± 0.11
**20**	0.1 ± 0.3	0.25 ± 0.21		1.0 ± 1.1	0.18 ± 0.09

This table presents the mean ± standard deviation of the sedatives: isoflurane (measured as mean alveolar concentration, MAC) for the isoflurane-group, propofol for the IV-group, and sufentanil for both groups, in relation to the treatment day.

Supplementary detailed analysis of organ function over time

Renal function

The renal function was monitored by creatinine and urea and showed no differences between the groups (creatinine: p=0.35; urea: p=0.58). Both time courses are presented in Supplementary Figure 4.

Figure S4. Time course of renal function parameters.

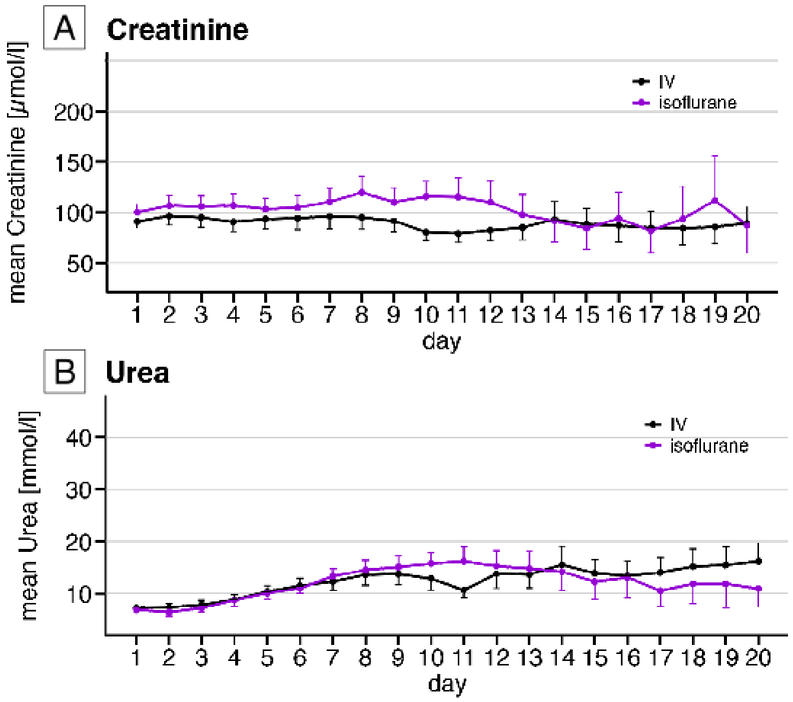

No. of patients

**Isoflurane**	36	36	34	30	30	29	29	26	25	22	19	17	15	14	11	11	11	10	9	9
**IV**	43	43	41	39	37	36	31	30	30	25	24	19	19	15	14	13	12	11	10	9

The figure displays daily mean levels of creatinine (upper panel, A) and urea (lower panel, B), with the isoflurane-group in violet and the IV-group in black. For each group, daily means and standard deviations across all patients are shown for the first 20 days, as patient numbers beyond this point were insufficient for reliable analysis.

Liver function

The liver function was monitored using GGT, AST, bilirubin and also PT and compared between the groups. GGT shows a statistically significant increase over the first 20 days, without differences between the groups either in the absolute values and in the time course (p= 0.69). AST is also significantly increased with no differences between the groups for the absolute values or the time course (p=0.09). Bilirubin is also increasing, where the increase is slightly lower in the isoflurane-group than the IV-group (p=0.01). All time courses are presented in Supplementary Figure 5.

Figure S5. Time course of the liver function parameters.

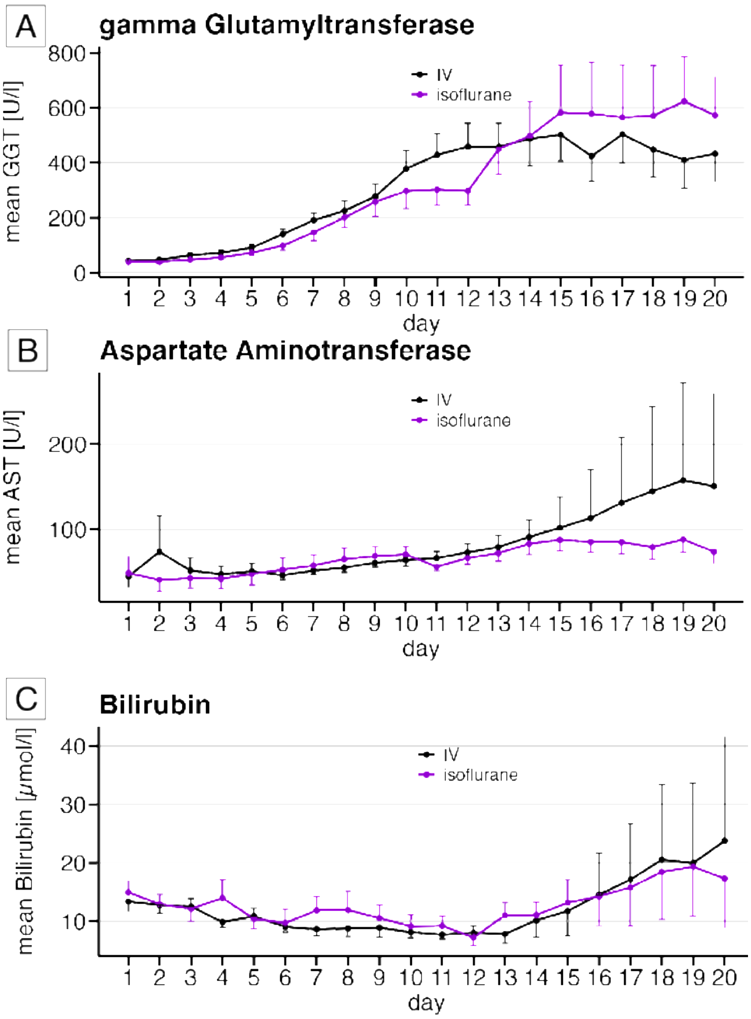

**Isoflurane**	36	36	34	30	30	29	29	26	25	22	19	17	15	14	11	11	11	10	9	9
**IV**	43	43	41	39	37	36	31	30	30	25	24	19	19	15	14	13	12	11	10	9

The figure displays daily mean levels of gamma glutamyltransferase (GGT, panel, A), aspartate aminotransferase (AST, panel B) and bilirubin (Panel C) with the isoflurane-group in violet and the IV-group in black. For each group, daily means and standard deviations

across all patients are shown for the first 20 days, as patient numbers beyond this point were insufficient for reliable analysis.

Hematopoietic system

The function of the hematopoietic system was monitored by the count of leucocytes, the haemoglobin concentration and the count of thrombocytes and compared between the groups. The leucocytes did not differ between groups for the absolute values or the time course (p=0.13). The concentration of haemoglobin significantly decreases over the first 20 days (<0.01) without differences in absolute values or time course between the groups (p=0.98). The thrombocytes increased significantly over time (p<0.01) but without differences between the isoflurane- and the IV-group (p=0.44). All time courses are presented in Supplementary Figure 6.

Figure S6. Time course of the hematopoietic system parameters.

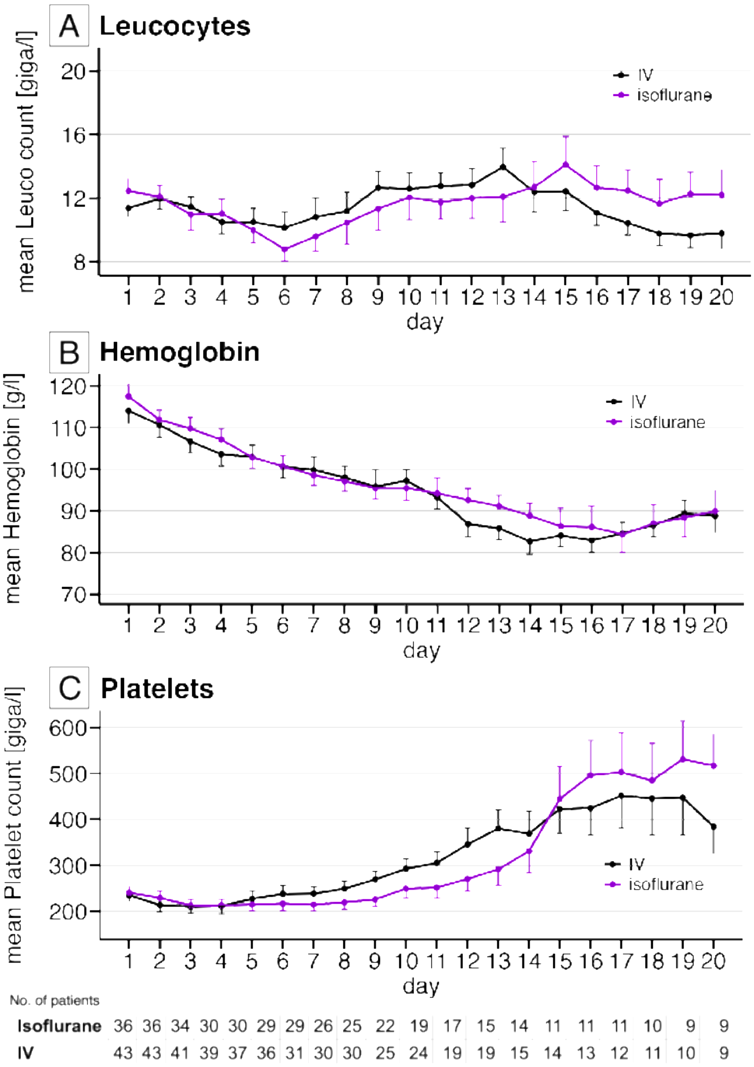

The figure displays daily mean levels of leucocyte counts (panel A), haemoglobin levels (panel B) and platelet counts (panel, C), with the isoflurane-group in violet and the IV-group in black. For each group, daily means and standard deviations across all patients are shown

for the first 20 days, as patient numbers beyond this point were insufficient for reliable analysis.

Carbon Dioxide

The level of paCO2 decreased over the first 20 days ($p<0.01$) but did not differ in absolute values or time course between the groups ($p=0.63$, Supplementary Figure 7).

Figure S7. Time course of the $paCO_2$.

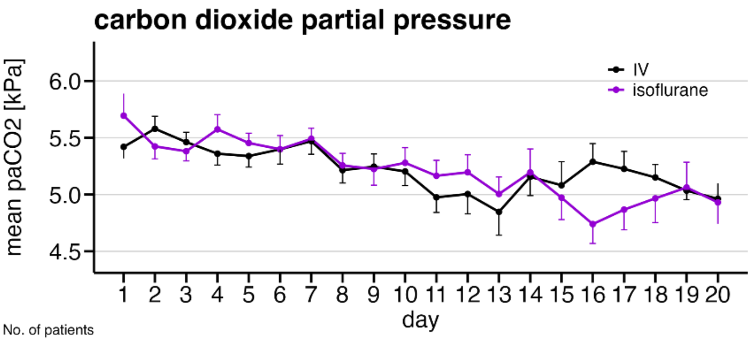

No. of patients

**Isoflurane**	36	36	34	30	30	29	29	26	25	22	19	17	15	14	11	11	11	10	9	9
**IV**	43	43	41	39	37	36	31	30	30	25	24	19	19	15	14	13	12	11	10	9

The figure displays daily mean levels of $paCO_2$ with the isoflurane-group in violet and the IV-group in black. For each group, daily means and standard deviations across all patients are shown for the first 20 days, as patient numbers beyond this point were insufficient for reliable analysis.
